# Supplementary material for: Differences in Reward Sensitivity between High and Low Problematic Smartphone Use Adolescents: An ERP Study
Source: Int J Environ Res Public Health. 2021 Sep 13;18(18):9603. doi: 10.3390/ijerph18189603 (PMC8470587; doi:10.3390/ijerph18189603)
Supplement: Supplementary file 1 [file ijerph-18-09603-s001.zip › ijerph-1280199-supplementary.pdf]

Supplementary Material S1

Mobile Phone Problematic Use Scale

(MPPUS-10, Foerster, Roser, Schoeni, & Röösli, 2015)

Listed below are a number of statements about your thoughts, feelings, and behaviors. Select the number that best matches your agreement or disagreement with each statement. Use the following scale, which ranges from 1 (not true at all) to 5 (extremely true). There are no right or wrong answers.

1. I have used my mobile phone to make myself feel better when I was feeling down.
2. When out of range for some time, I become preoccupied with the thought of missing a call.
3. If I don't have a mobile phone, my friends would find it hard to get in touch with me.
4. I feel anxious if I have not checked for messages or switched on my mobile phone for some time.
5. My friends and family complain about my use of the mobile phone.
6. I find myself engaged on the mobile phone for longer periods of time than intended.
7. I am often late for appointments because I'm engaged on the mobile phone when I shouldn't be.

8. I find it difficult to switch off my mobile phone.

9. I have been told that I spend too much time on my mobile phone.

10. I have received mobile phone bills I could not afford to pay.

Supplementary Material S2

In order to control the impact of demographic variables and individual differences in the level of sensation seeking and habitual use of emotional regulatory strategy (expression suppression and cognitive reappraisal), we conducted repeated measures ANOVA to exclude the impact of these variables on our results of ERPs. The results of the repeated measures ANOVA indicated that the main effects of these variables on the ERPs were not significant. Therefore, the demographic variables were not included in subsequent analyses. The impact of participants differences in the sensation seeking, emotion regulation and

For P3: gender: $F(1,45) = .018$, $p = .895$, $\eta_p^2 = .000$; only child: $F(1,45) = .666$, $p = .419$, $\eta_p^2 = .015$; father's education level: $F(1,45) = .014$, $p = .906$, $\eta_p^2 = .000$; mother's education level: $F(1,45) = .126$, $p = .724$, $\eta_p^2 = .003$; sensation seeking: $F(1,44) = 1.718$, $p = .197$, $\eta_p^2 = .038$; cognitive reappraisal: $F(1,44) = .393$, $p = .534$, $\eta p^2 = .009$; expression suppression: $F(1,44) = 1.007$, $p = .321$, $\eta p^2 = .022$.

For FRN: gender: $F(1,45) = 1.628$, $p = .209$, $\eta_p^2 = .035$; only child: $F(1,45) = .005$, $p = .946$, $\eta_p^2 = .000$; father's education level: $F(1,45) = .021$, $p = .884$, $\eta_p^2 = .000$; mother's education level: $F(1, 45) = .245$, $p = .623$, $\eta_p^2 = .005$; sensation seeking: $F(1,44) =.592$, $p = .446$, $\eta_p^2 = .013$; cognitive reappraisal: $F(1,44) = .002$, $p = .965$, $\eta p^2 < .001$; expression suppression: $F(1,44) = .810$, $p = .373$, $\eta_p^2 = .018$.
